# Gender and Age Differences in Meal Structures, Food Away from Home, Chrono-Nutrition, and Nutrition Intakes among Adults and Children in Tanzania Using a Newly Developed Tablet-Based 24-Hour Recall Tool

**DOI:** 10.1093/cdn/nzac015

**Published:** 2022-02-08

**Authors:** Ramya Ambikapathi, Imani Irema, Isaac Lyatuu, Bess Caswell, Dominic Mosha, Stella Nyamsangia, Lauren Galvin, Ally Mangara, Morgan Boncyk, Savannah L Froese, Cristiana K Verissimo, Julieth Itatiro, Victoria Kariathi, Patrick Kazonda, Medina Wandella, Wafaie Fawzi, Japhet Killewo, Mary Mwanyika-Sando, George PrayGod, Germana Leyna, Crystal Patil, Nilupa S Gunaratna

**Affiliations:** Departments of Public Health and Nutrition Science, Purdue University, West Lafayette, IN, USA; Africa Academy of Public Health, Dar es Salaam, Tanzania; Africa Academy of Public Health, Dar es Salaam, Tanzania; Western Human Nutrition Research Center, Agricultural Research Service, US Department of Agriculture, Davis, CA, USA; Africa Academy of Public Health, Dar es Salaam, Tanzania; Africa Academy of Public Health, Dar es Salaam, Tanzania; Global Communities, Dar es Salaam, Tanzania and Silver Spring, MD, USA; Department of Epidemiology and Biostatistics, Muhimbili University of Health and Allied Sciences, Dar es Salaam, Tanzania; Departments of Public Health and Nutrition Science, Purdue University, West Lafayette, IN, USA; Departments of Public Health and Nutrition Science, Purdue University, West Lafayette, IN, USA; Departments of Public Health and Nutrition Science, Purdue University, West Lafayette, IN, USA; Tanzania Food and Nutrition Center, Dar es Salaam, Tanzania; Tanzania Food and Nutrition Center, Dar es Salaam, Tanzania; Department of Epidemiology and Biostatistics, Muhimbili University of Health and Allied Sciences, Dar es Salaam, Tanzania; Tanzania Food and Nutrition Center, Dar es Salaam, Tanzania; Department of Global Health, Harvard TH Chan School of Public Health, Boston, MA, USA; Department of Epidemiology and Biostatistics, Muhimbili University of Health and Allied Sciences, Dar es Salaam, Tanzania; Africa Academy of Public Health, Dar es Salaam, Tanzania; National Institute for Medical Research, Mwanza, Tanzania; Department of Epidemiology and Biostatistics, Muhimbili University of Health and Allied Sciences, Dar es Salaam, Tanzania; Tanzania Food and Nutrition Center, Dar es Salaam, Tanzania; Department of Women, Children and Family Health Science, University of Illinois Chicago, Chicago, IL, USA; Departments of Public Health and Nutrition Science, Purdue University, West Lafayette, IN, USA

**Keywords:** tablet, open data kit, dietary data, nutrient intakes, Tanzania, diets, children, adults

## Abstract

**Background:**

In many regions of the world, little is known about meal structures, meal patterns, and nutrient intake because the collection of quantitative dietary intake is expensive and labor-intensive.

**Objectives:**

We describe the development and field feasibility of a tablet-based Tanzania 24-h recall tool (TZ-24hr-DR) and dietary intakes collected from adults and children in rural and urban settings.

**Methods:**

Using the Tanzanian food-composition table, the TZ-24hr-DR tool was developed on an Android platform using the Open Data Kit. The module provides food lists, meal lists, ingredient lists, quantity and amount consumed, breastfeeding frequency, and a recipe feature. Similar to the USDA Automated Multiple Pass Method, this TZ-24hr-DR contains review features such as time in-between meals, a summary of meals, and portion sizes.

**Results:**

Dietary intake using TZ-24hr-DR was collected among *1*) 845 children 0–18 mo of age enrolled in the Engaging Fathers for Effective Child Nutrition and Development in Tanzania (EFFECTS) trial (ClinicalTrials.gov identifier: NCT03759821) in Mara, Tanzania, and *2*) 312 adult families from the Diet, Environment, and Choices of positive living (DECIDE) observational study in peri-urban Dar es Salaam. Interviewers were trained on paper-based methods with food models and tablet-based collection. Conversion to nutrient intake was readily linked and accessible, enabling rapid review and analysis. Overall, 2158 and 8197 dietary meal records were collected from the DECIDE study and EFFECTS trial, respectively. Among adults, 63% of men and 92% of women reported eating at home, and there were differences in protein, fat, and zinc. Food consumed outside the home typically occurs for the first 2 meals. Children's intake of nutrients increased with age; however, median micronutrient intakes for calcium, iron, zinc, and vitamin A remained below recommended nutrient intakes.

**Conclusions:**

The TZ-24hr-DR is a field- and user-friendly tool that can collect large samples of dietary intakes. Further validation is needed. The tool is available freely for research purposes and can be further adapted to other contexts in East Africa.

## Teaser

A tablet-based quantitative dietary intake tool was developed for Tanzania and used to survey 2 populations: rural children and peri-urban adults living with HIV.

## Introduction

Globally, dietary risk factors account for one-quarter of all deaths (1). In addition, there is an increasing burden from over- and undernutrition due to drastic shifts in food environments, consumption patterns, migration, and demographic transition in low- and middle-income countries (LMICs) (2–8). However, little is known about dietary patterns in LMICs, particularly meal structures, the timing and location of meal patterns, and nutrient intakes (9). To address the shifting nutrition profiles, public health programs require data capturing dietary intake and consumption patterns of individuals and populations that are easy to collect and reliable, especially assessing dietary data over time (9). However, collecting these data using quantitative dietary intake, particularly the 24-h dietary methodology, is expensive and labor-intensive for 2 main reasons: first, collecting dietary recalls requires interviewing and probing skillsets, quantitative estimation, and general knowledge of recipes and cooking practices in the local context (10), and second, the processing of dietary data to estimate portions consumed and nutrient intakes requires up-to-date food-composition databases and often requires staff with nutrition training.

These 2 labor-intensive steps have primarily reduced the availability of quantitative dietary data collection in LMICs. The availability and use of digital tools such as tablets or smartphones combined with the availability and use of open-source software such as Open Data Kit (ODK) have enabled adopting the collection of dietary recalls in LMICs. We have identified 7 digital dietary recall tools that have been used in LMICs so far, including the tool from this current paper (11–16). Here, we focus on tablet-based rather than computer or online self-reported tools for the feasibility of data collection in the field among vulnerable populations who may not have the education and familiarity for self-reported instruments. Out of these 7 identified tools, 3 tools are built using an open-source platform, 2 include recipe features, and all have used aids, whether food models or utensils or photographs, in the recall. More recently, the International Dietary Data Expansion Project has established 9 criteria for evaluating whether the 24-h-recall digital dietary data collection is optimal for scale-up and use in nutrition programs (17). These criteria include use of multiple-pass interview methods, being interview administered, having photo aids for portion-size estimation, links to a food-composition database, ability to use both paper and electronic data collection, field feasibility (running offline), adaptability across different contexts, scalable for national surveys, ease of use, and finally, low cost to adopt (17). All the identified seven dietary assessment tools use multiple-pass methods, offline data collection features, and are interview administered. Only half of the tools have pictorial aids, while one-third were tested across different contexts. More importantly, only 3 tools are available freely (either upon request or available online).

The main purpose of this paper is to describe the development and field feasibility of a tablet-based Tanzania 24-h dietary recall tool (TZ-24hr-DR) and assess dietary intakes collected using the TZ-24hr-DR tool from adults and children in urban and rural Tanzanian settings. Finally, dietary intakes collected from this tool are compared with other relevant studies conducted in the same settings (12). We have added several innovative features, such as the summary of meals, portion size, timing of dietary intakes, ability to add recipes, and location of meal consumption, to reduce data collection and estimation error. Here we provide information on meal structures, chrono-nutrition, meals away from home, and nutrient intakes in 2 rapidly changing food environment settings in Tanzania.

## Methods

### Study setting

The TZ-24hr-DR was used in 2 study populations (adults and children) in Tanzania. First, this tool was used in an observational cohort called the Diet, Environment, and Choices of positive living (DECIDE) study, which evaluates personal and external food environments among adult people living with HIV (PLHIV) and an adult family member of theirs (18). Data from the first wave of data collection are presented here. The DECIDE cohort is set in the Dar es Salaam Urban Cohort Study, which is a low-income community right outside of the commercial capital of Tanzania (19). Second, TZ-24hr-DR was used in a baseline evaluation of Engaging Fathers for Effective Child Nutrition and Development in Tanzania Study (EFFECTS; ClinicalTrials.gov Identifier: NCT03759821), a cluster-randomized trial among rural agrarian families with children 0–18 mo of age in the northern Mara region, Tanzania. The EFFECTS trial tested the impact of father engagement (in addition to mothers) to improve child nutrition and development. The National Institute for Medical Research in Tanzania provided ethical approvals for both Tanzanian studies. In addition, the DECIDE study had Institutional Review Board (IRB) approvals from Purdue University, and the EFFECTS study had IRB approvals from Harvard University, through which Purdue has a reliance agreement. Informed consent was provided by adults enrolled in the DECIDE study and by the caregivers (both parents) for collecting dietary data among children enrolled in the EFFECTS study. Participants did not receive any monetary compensation for the collection of data.

### Enrollment of the study participants

Enrollment in the DECIDE study has been described elsewhere (18). Briefly, 2600 participants were screened at community clinics in 2019 and 396 were eligible to participate in the DECIDE study. PLHIV participants were eligible to participate if they were above 18 y of age, provided informed consent to participate in both rounds of the study, and lived in the study area. The adult family member also provided informed consent. Out of 396 eligible participants, 70 were not available for interviews or moved away before the first round of data collection. Out of the remaining 326 participants, 14 had missing 24-h recalls, so 312 PLHIV participants, and their family members when available, were included from the DECIDE study. Enrollment in the EFFECTS study has also been described elsewhere (20). Briefly, households from 80 villages in 2 districts in the Mara region of Tanzania were enrolled in a cluster-randomized controlled trial in 2019. Households were eligible to participate if they had a child aged 0–18 mo at enrollment, both parents consented to participate in interventions (except for those in control arms) and 3 annual evaluations, the household intended to stay in the study setting for the duration of the study, and the father would be present in the household for at least 10 mo of the year. Overall, 960 households were enrolled; however, 30 households were lost to follow-up before the first round of data collection and were replaced using the same eligibility criteria. For these analyses, we included the 88% of children who had consumed non–breast-milk foods in the previous day at the time of baseline data collection.

### Development of the TZ-24hr-DR tool

Using the Tanzanian food-composition table (FCT), the TZ-24hr-DR tool was developed on an Android platform using ODK (20, 21). This development entailed revising a paper version of the 24-h recall questionnaire into a standard Excel form called the XLSForm, which was then converted into an ODK format called the XForm. The XLSForm included food item files from Tanzania FCT and incorporated quality, flow control, and skip patterns as the interviewer selected the list of menus nested under each choice. Flow control refers to the order through which the interviewer recorded information, and skip pattern refers to a sequence of choices based on previous answers. For example, the selection of cereal food group will produce a list of ingredients and meals with cereal as an ingredient from the Tanzanian FCT, which includes a list of 31 food items (such as infant cereal, sorghum porridge, etc.) (20). The flow control and skip patterns mimicked paper-based dietary recall methods and were iteratively optimized based on feedback from the nutritionists and interviewers, thus improving the overall quality of data collection.

The training was conducted in 2 phases among interviewers who had completed high school education and had experience conducting public health surveys in these communities. First, interviewers were trained using paper-based methods to become acquainted with the concept of a 24-h dietary recall interviewer form. Then, they were oriented on interviewing techniques, probing, free listing activities, and meals consumed in the previous 24 h. After that, detailed information on ingredients, portion sizes, and serving sizes was collected for each meal. The second phase of the training included an orientation on collecting 24-h recall using a tablet. These training sessions on tablet data collection were done by a facilitator (author DM) and a nutritionist (authors SN, RA, and CP) and used examples to show how to use the tool. Later, fieldworkers practiced among themselves and did a short pilot training in the community before deployment.

Fieldworkers were trained with 3 interview aids: serving size utensils, food lists, and a notebook for free listing activities and meals. Reference serving size aids included 7 utensils: bowl, cup, tumbler, plate, tablespoon, teaspoon, and saucer (see **Supplemental Figure 1**). Since most fieldworkers were not familiar with how meals were categorized under food groups, the data-collection process was slow initially. This warranted us to give each interviewer a food group and meal list and additional training on the most consumed foods for each study context. Reference lists included food items categorized by food groups (5 pages) taken from the Tanzanian FCT and commonly consumed foods in each setting (1–2 pages). Finally, the trained interviewers were also provided with paper notebooks for free listing activities and meals during the interview.

### Phase 1: dietary data collection

**FIGURE 1:**
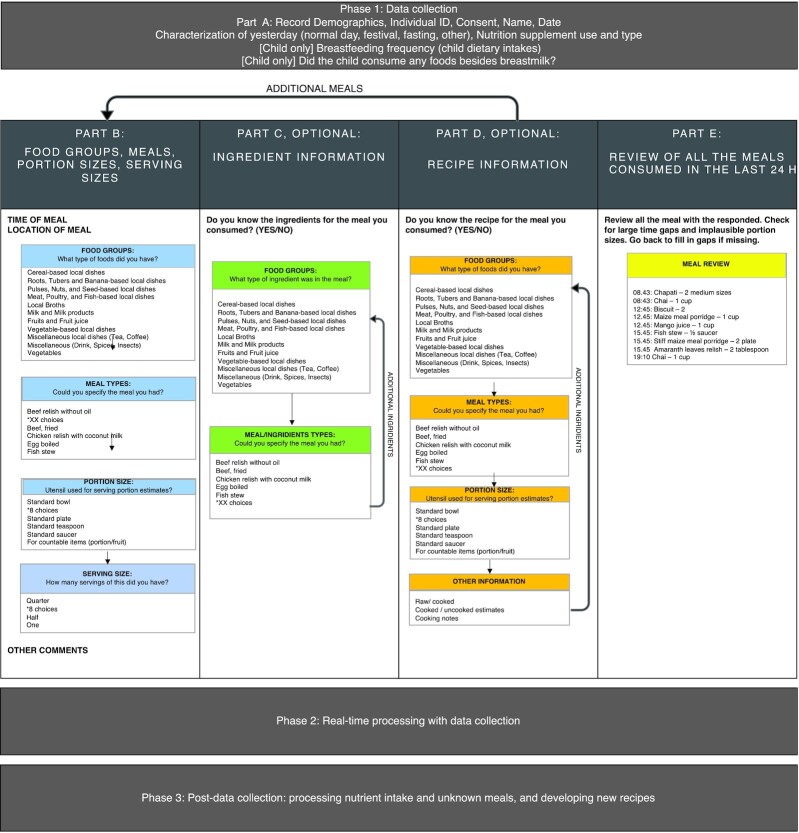
Flow of TZ-24hr-DR tool from demographics, food consumption, ingredient information, and recipe information. Asterisks indicate there were many choices. TZ-24hr-DR, Tanzania 24-h recall tool.

**Figure 1** illustrates the logic and dietary data-collection flow in the tablet. First (part A), we collected information on participants’ demographics, characterization of consumption day (typical, holiday, fasting, etc.), and nutritional supplement use. In part B, information on the consumed food was collected. The food consumption loops were structured based on the Tanzanian FCT. A meal is defined as any set of food, snacks, and beverages consumed at the same time. The interviewer selected the times and location of each meal, followed by the food group of each, then meal list, details on the meals with ingredient lists, portion sizes, and finally serving size. There was also the option to enter unknown meals as “other” in part B. Unknown meals refer to food items not pre-populated by the Tanzanian FCT. Then, in part C, we asked the participant about the ingredients of the consumed meal because it was common for people to eat take-out meals and prepared meals in urban settings. Listing ingredients on the consumed meal was an optional question. The information on ingredients helped us to correct for the appropriate FCT code. For example, there are 2 kinds of samosas, one with meat and one with potatoes, and they vary distinctly in terms of nutrients and energy. In part D, we asked the participant if they knew the recipe of the meals. The recipe feature allowed the interviewer to collect detailed information such as cooked and uncooked weight and a free-text feature for notes on the cooking method. There was a free-text option called “other comments” for each meal where the interviewer was instructed to collect additional information on feeding, brand, and parts of the meal consumed. Some examples include siblings feeding the baby even though the mother prepared the meal or consuming only the broth, not the meat portion of fish stew.

At the end of each meal-recipe (part B to D) loop, interviewers were asked if they wanted to add a meal or finish the survey for review. If the survey is completed, the TZ-24hr-DR provides a summary of all the meals consumed (part E). This summary included timing of meals, review of meals, and portion size consumed in the previous day for the interviewer to review any significant time gaps, implausible portion sizes (e.g., 8 cups of cooked rice for a 13-mo child), and meals (e.g., strawberry juice in remote Mara region is not possible because strawberries are not available here).

Other dietary information, such as nutritional supplement use, breastfeeding frequency, and family diets (e.g., DECIDE study collected data from 2 members of the same household), can also be captured with this tool. Breastfeeding was also entered as a meal at each specific time the mother fed the child. Additional quality checks were included on breastfeeding—for example, the breastfeeding frequency had to match the number of breastfeeding “meals” entered. For example, if the interviewer entered 6 for breastfeeding frequency at the beginning of the survey, they also must enter 6 breastfeeding meals with timing and location.

### Phase 2: real-time processing of dietary recalls

**FIGURE 2:**
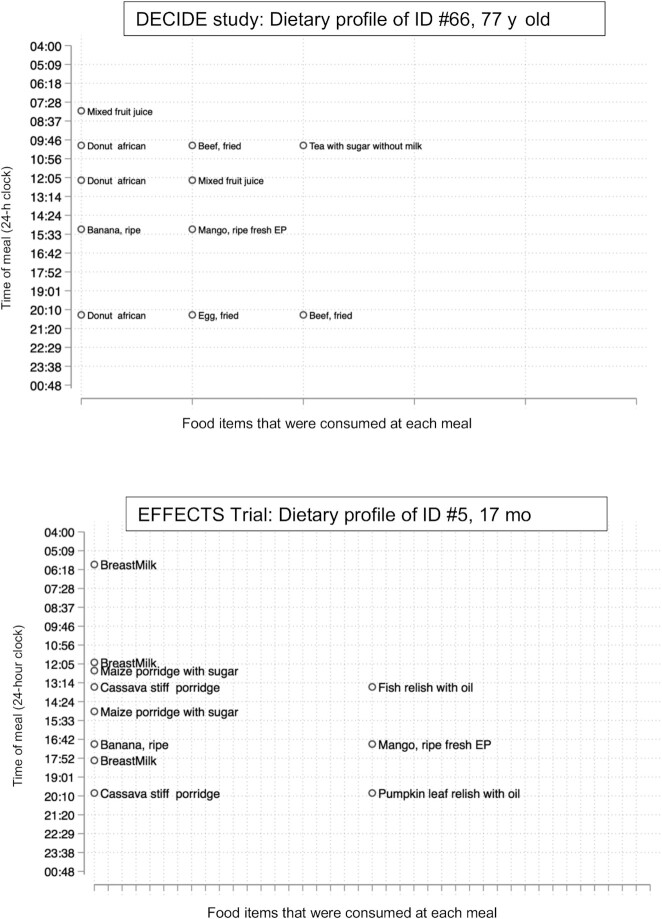
Sample individual dietary profiles were created daily based on uploaded data (top panel: DECIDE study; bottom panel: EFFECTS Trial). DECIDE, Diet, Environment, and Choices of positive living; EFFECTS, Engaging Fathers for Effective Child Nutrition and Development in Tanzania. EP refers to edible portion.

Data collection for the first round of the DECIDE study among 312 PLHIV adults and 214 family members of PLHIV was conducted between February and June 2019. We report dietary intakes from the 312 PLHIVs’ dietary intake (family members’ dietary intakes are not included here). The baseline evaluation for the EFFECTS trial was conducted from December 2018 to May 2019. In the EFFECTS trial, dietary recalls from children were collected from the primary caregiver responsible for preparing food and feeding the child (mother). Primary caregivers, usually mothers, were queried for the dietary recall for her child enrolled in the EFFECTS trial. Since the data were collected and transmitted readily to the server with the availability of the 3G mobile network, we were able to do real-time processing and create daily reports to review any issues with data collection. These included missing forms, the timing of first meals, frequently consumed meals by meal order (breakfast, lunch, dinner, and any other meals in-between including snacks and beverages), breastfeeding frequency (sample report from the EFFECTS trial is included in **Supplemental Report 1**), and creation of dietary profiles for everyone (**Figure 2**). Any issues, such as implausible values or timings or food items, raised in the automated report were relayed to the on-site nutritionist and interviewers.

### Phase 3: post–data collection: processing nutrient intakes

Each dietary recall from an individual was collected in 2 datasets, which were sent to the servers on the same day: *1*) first, a demographics dataset that had 1 record per participant per day, which included information collected in part A of Figure 1, and *2*) a secondary meal dataset, which had multiple records of meals per participant per day (part B to D). Each meal record was easily linked to the Tanzanian FCT because it used the same code. Users can also enter unknown meals by code “9999,” along with the description of the meals. These unknown meals were manually checked for any corresponding codes in the Kenyan FCT since they were not found in the Tanzanian (22). If a similar code was not found in the Tanzanian FCT or the Kenyan FCT, a recipe using the user-provided input on ingredients and/or recipe instruction was created and assigned to each specific unknown meal (code: “8888”; see next section on unknown/recipe analysis). Then, these changes were made in Stata files to be reviewed additionally by trained nutritionists for appropriate assignments. After all the codes were assigned, they were linked to the updated portion-size databases to estimate the amount consumed. Breastfeeding was not included in the nutrient estimates.

For these 2 study settings, updated portion size included 88 new estimates (e.g., weight estimates for various sizes of watermelon slices that are typically sold, developed by author SN). Then, the amount consumed for each meal was multiplied by nutrient intakes for that specific meal code to yield nutrient intakes. Finally, the nutrient intakes were processed, assigned, and reported using Stata to record and review all changes.

There were 670 unknown meal records from the EFFECTS baseline data collection and 222 unknown meal records from the DECIDE first wave. A food item was flagged as unknown when a code did not exist in the Tanzanian FCT. In these cases, a description and list of ingredients were reviewed for each meal to assess whether an existing meal ID from the Tanzanian or Kenyan FCTs could be assigned. If a meal was completely new (i.e., not in the Tanzanian or Kenyan FCTs), recipes were created based on recipes available and online searches. For each created recipe, the web source was documented along with the portions of each ingredient. Most ingredients were found in the Tanzanian or Kenyan FCTs. If the ingredient did not exist in one of these FCTs (e.g., octopus), the ingredient nutrition information was obtained from USDA FoodData Central (23). Nutrient retention factors for locally prepared recipes were not available and were not applied in the recipes. Nutrients for new recipes were calculated per 100 g. Overall, 28 new recipes were developed for these 2 study settings (**Supplemental Table 1**). In addition, there was an option for the interviewer to enter recipe information, even for an existing meal code from Tanzanian FCT, to examine if standard recipes are changed for personal preferences. In total, 825 recipes were collected from the respondents.

## Results

**Tables 1**and **2** summarize the demographic characteristics of both study populations. From the DECIDE study, dietary data on 312 adult PLHIV (75.3% female) participants living in peri-urban Dar es Salaam were collected, while data on 845 children (49.7% female) under 2 y of age were collected from the EFFECTS trial. For DECIDE participants, the median age was 41 y, and the median education was seventh grade. One-third of the DECIDE households were food secure, while only 11% of the households were food secure in the EFFECTS trial. In the EFFECTS trial, most of the children aged 11 mo (82%) had breast milk in their diets. Wood was the common cooking fuel in the rural EFFECTS trial (95%), while charcoal was common in the urban DECIDE study (74%). We present results by gender for adults (DECIDE study) and by age groups for children (EFFECTS trial, when possible) because adults’ diets vary notably by gender, while child diets vary as they age in their early childhood years.

**FIGURE 4:**
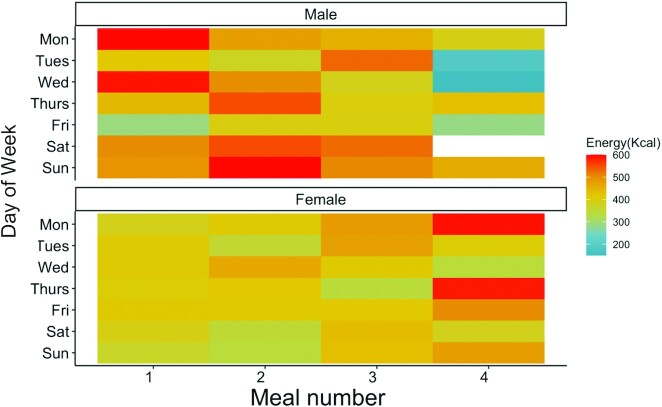
Mean energy intake by the meal of the day by gender from the DECIDE study (PLHIV adults). DECIDE, Diet, Environment, and Choices of positive living; PLHIV, persons living with HIV.

**TABLE 1:** Background characteristics of the DECIDE study (by gender)^1^

	DECIDE study (PLHIV adults)		
	Male (*n* = 77)	Female (*n* = 235)	Total (*n* = 312)
Age, y	44 (37, 50)	40 (32, 47)	41 (33, 48)
Are you the head of the household? (%)			
Yes	72.7 (56)	37.9 (89)	46.5 (145)
Participant education (%)			
Up to seventh grade	75.3 (58)	77.4 (182)	76.9 (240)
Above seventh grade	24.7 (19)	22.6 (53)	23.1 (72)
What is the main fuel used for cooking? (%)			
Gas (industrial)	15.6 (12)	13.6 (32)	14.1 (44)
Paraffin	11.7 (9)	2.1 (5)	4.5 (14)
Charcoal	68.8 (53)	75.3 (177)	73.7 (230)
Firewood	3.9 (3)	8.9 (21)	7.7 (24)
BMI, kg/m^2^	21.9 (20.2, 24.9)	23.7 (20.7, 28.8)	23.2 (20.5, 27.6)
Household Food Insecurity Access (%)			
Food secure	31.2 (24)	27.2 (64)	28.2 (88)
Mildly food insecure	5.2 (4)	9.8 (23)	8.7 (27)
Moderate food insecurity	27.3 (21)	29.8 (70)	29.2 (91)
Severe food insecurity	36.4 (28)	33.2 (78)	34.0 (106)

1 Values are *n* (%) or median (IQR). DECIDE, Diet, Environment, and Choices of positive living; PLHIV, persons living with HIV.

**TABLE 2:** Background characteristics of the EFFECTS trial (by age groups)^1^

	EFFECTS trial (children)				
	0–5 mo (*n* = 83)	6–12 mo (*n* = 360)	13–18 mo (*n* = 262)	19–24 mo (*n* = 140)	Total (*n* = 845)
Age, y	3.0 (3.0, 4.0)	9.00 (7.0, 10.0)	15.0 (13.0, 17.0)	19.0 (18.0, 20.0)	12.0 (8.0, 16.0)
Head of household education (%)					
Up to seventh grade	88.6 (70)	87.8 (294)	85.0 (209)	88.7 (118)	87.1 (691)
Above seventh grade	11.4 (9)	12.2 (41)	15.0 (37)	11.3 (15)	12.9 (102)
Breastfeeding in the previous 24 h (%)					
No	0.0 (0)	2.8 (10)	20.1 (51)	59.9 (82)	17.1 (143)
Yes	100.0 (83)	97.2 (350)	79.9 (203)	40.1 (55)	82.9 (691)
Common cooking fuel (%)					
Wood	94.9 (75)	95.9 (327)	94.4 (235)	92.6 (126)	94.8 (763)
Charcoal	5.1 (4)	3.8 (13)	5.6 (14)	7.4 (10)	5.1 (41)
Other	0.0 (0)	0.3 (1)	0.0 (0)	0.0 (0)	0.1 (1)
Grid electricity (%)					
No	86.1 (68)	91.8 (313)	91.6 (228)	94.9 (129)	91.7 (738)
Yes	13.9 (11)	8.2 (28)	8.4 (21)	5.1 (7)	8.3 (67)
Weight-for-age *z*-score	−0.23 (−1.15, 0.57)	−0.47 (−1.24, 0.22)	−0.72 (−1.44, −0.09)	−0.77 (−1.65, −0.11)	−0.59 (−1.28, 0.11)
Household Food Insecurity Access (%)					
Food secure	3.6 (3)	12.5 (45)	14.9 (39)	10.0 (14)	12.0 (101)
Mildly food insecure	15.7 (13)	13.1 (47)	15.3 (40)	19.3 (27)	15.0 (127)
Moderate food insecurity	22.9 (19)	26.7 (96)	21.4 (56)	18.6 (26)	23.3 (197)
Severe food insecurity	57.8 (48)	47.8 (172)	48.5 (127)	52.1 (73)	49.7 (420)

1 Values are *n* (%) or median (IQR). EFFECTS, Engaging Fathers for Effective Child Nutrition and Development in Tanzania.

A total of 2158 and 8197 dietary meal records were collected from the DECIDE study and EFFECTS trial, respectively. In **Tables 3** and **4**, descriptions of meals and the location of food consumption from both studies are shown. DECIDE participants had a median of 7 meal records (IQR: 5, 8) and 3 meals/d (IQR: 3, 4). Sixty-three % of the male participants reported eating meals at home compared with 92% of women. Less than 1% of adult PLHIV participants reported supplement use. Children's meal frequency decreased as they aged, from consuming 9 meals (including breastfeeding) under 12 mo of age to 6 meals by the time they reached 19–24 mo of age. By 19–24 mo, only 39% of the mothers reported breastfeeding on the previous day.

**TABLE 3:** Description of typical meals/foods consumed by participants in the DECIDE study^1^

	DECIDE study (PLHIV adults)			
	Male (*n* = 77)	Female (*n* = 235)	Total (*n* = 312)	*P*
Number of records per day	7 (6, 8)	7 (5, 8)	7 (5, 8)	0.89
Meal frequency	3 (3, 3)	3 (3, 4)	3 (3, 4)	0.81
What best characterizes yesterday?				0.83
Typical day	98.7 (75)	98.3 (237)	98.4 (312)	
Fasting day	1.3 (1)	1.7 (4)	1.6 (5)	
Did you take any supplements yesterday?				0.082
Yes	2.6 (2)	0.4 (1)	0.9 (3)	
Where was this meal (food/drink) consumed?				<0.001
Home	62.9 (330)	92.4 (1519)	85.2 (1849)	
Work	5.7 (30)	2.1 (35)	3.0 (65)	
At a friend's house	1.1 (6)	0.5 (8)	0.6 (14)	
Roadside vendor	5.0 (26)	1.1 (18)	2.0 (44)	
*Mamalishe* (women prepared food vendors)	10.5 (55)	0.6 (10)	3.0 (65)	
Restaurant	10.9 (57)	2.1 (34)	4.2 (91)	
Hotel	2.1 (11)	0.4 (6)	0.8 (17)	
Other	1.9 (10)	0.9 (14)	1.1 (24)	

1 Values are *n* (%) or median (IQR). DECIDE, Diet, Environment, and Choices of positive living; PLHIV, persons living with HIV.

**TABLE 4:** Description of typical meals/foods consumed by participants in the EFFECTS trial^1^

	EFFECTS trial (children)					*P* (adjusted for village clustering)
	0–5 mo (*n* = 83)	6–12 mo (*n* = 360)	13–18 mo (*n* = 262)	19–24 mo (*n* = 140)	Total (*n* = 845)	
% Children breastfed yesterday	100.0 (83)	97.5 (351)	77.9 (204)	38.6 (54)	81.9 (692)	<0.001
Breastfeeding frequency	7 (5, 8)	5 (4, 7)	4 (2, 5)	0 (0, 3)	5 (3, 6)	<0.001
Meal frequency (including breastfeeding)	9 (7, 11)	9 (7, 11)	8 (6, 10)	6 (4, 8)	8 (6, 10)	<0.001
What best characterizes yesterday?						
Typical day	96.4 (80)	96.7 (348)	97.3 (255)	97.1 (136)	96.9 (819)	0.764
Holiday/special day/Other	3.6 (3)	3.3 (12)	2.7 (7)	2.9 (4)	3.1 (26)	

1 Values are *n* (%) or median (IQR). EFFECTS, Engaging Fathers for Effective Child Nutrition and Development in Tanzania.

**Tables 5** and **6** summarize the most frequently consumed foods by adults and children in the DECIDE and EFFECTS studies, disaggregated by gender and age groups, respectively. The most consumed food items across both studies were stiff maize porridge, tea, rice boiled in water with oil, beans, beef relish, and green leafy relish. In the DECIDE study, the commonly consumed meat items were fish and beef relish (and beef broth for men). Ultra-processed foods such as African donuts (made of highly processed food such as flour, sugar, oil) and carbonated drinks were also frequently consumed, and these were often consumed outside the house. The most consumed meals outside of the home were carbonated sodas, chapati with oil, rice boiled in oil, tea, and fried potato chips. In the EFFECTS trial, meal consumption patterns by age group of children revealed an interesting trend. Commonly consumed foods for children under 5 mo of age were cow milk and variations of soft porridge made of flour, maize, cassava, and finger millet porridge (watery consistency) with sugar and/or milk. Very few dietary records showed intake of legumes (in relish form), fruits (papaya and mango), and meat (fish and beef relish) among children. Among children aged 6–12 mo, diets began to include *ugali* (stiff porridge, has a solid consistency) and more fish either as *dagaa* (small, dried fish) relish or as a stew. The most consumed fruit for this age group was mango. Among children 12–18 mo of age, rice, green-leafy vegetables such as amaranth leaves relish, tea with sugar and milk, and kidney bean relish are introduced, and higher quantities of fish. By 19–24 mo of age, stiff and soft porridge, fish relish, and some additional fruits were part of the diet. There was also a more significant variety of green-leafy vegetables in the diet for older children. Median meal frequency without breastfeeding increased from 2 meals (IQR: 2, 3) for children under 6 mo of age to 5 meals (IQR: 3, 6) for children 18–25 mo of age.

**TABLE 5:** Most frequently consumed food by adults and children in DECIDE and EFFECTS studies^1^

DECIDE female PLHIV		DECIDE male PLHIV	
Most frequently consumed foods	Frequency (*n* = 1614)	Most frequently consumed foods	Frequency (*n* = 544)
1. Maize *ugali* (stiff porridge)	230	1. Maize *ugali* (stiff porridge)	66
2. Tea without milk, with sugar	181	2. Tea without milk, with sugar	47
3. Rice boiled with oil	123	3. Rice boiled with oil	39
4. Beans, kidney, mature, boiled without salt	59	4. Chapati with oil	26
5. Green leaf, medium, relish without oil	51	5. Beef relish with oil	25
6. Donut-African	45	6. Beans, kidney, mature, boiled without salt	23
7. Chapati with oil	41	7. Carbonated, beverage, Coca-Cola	21
8. Fish relish with oil	39	8. Donut-African	16
9. Cassava, fried	34	9. Green leaf, medium, relish without oil	12
10. Carbonated, beverage, Coca-Cola	33	10. Tea with milk and sugar	11
11. Beef relish with oil	32	11. Banana, ripe	9
12. Sweet potato leaf	32	12. Beef broth with oil	9
13. Fish, fried	27	13. Bread, white	9
14. Banana, ripe	25	14. Sweet potato leaf	9
15. Fish relish with coconut milk	23	15. Fish relish with oil	8
16. Small dried fish, fried	23	16. Kidney bean relish with oil	8
17. Potato chips, fried	19	17. Potato leaf relish with oil	8
18. Rice with coconut milk	19	18. Fish, fried	7
19. Small dried fish with tomatoes and oil	19	19. Potato chips, fried	7
20. Bread, white	18	20. Cabbage, onion salad	6

1 Numbers represent frequency out of total meals across all dietary recalls. DECIDE, Diet, Environment, and Choices of positive living; EFFECTS, Engaging Fathers for Effective Child Nutrition and Development in Tanzania; PLHIV, persons living with HIV.

**TABLE 6:** Most frequently consumed food by children in EFFECTS trial^1^

0–5 mo (*n *= 210)		6–12 mo (*n *= 1634)		13–18 mo (*n *= 1576)		19–24 mo (*n *= 911)	
Most frequently consumed food	Frequency	Most frequently consumed food	Frequency	Most frequently consumed food	Frequency	Most frequently consumed food	Frequency
Milk whole, 3.25% milk fat	81	Maize *ugali* (stiff porridge)	162	Maize *ugali* (stiff porridge)	162	Maize *ugali* (stiff porridge)	102
Mix flour porridge with sugar	41	Mix flour porridge with sugar	120	Mix flour porridge with sugar	120	Maize porridge without sugar and milk	84
Maize porridge with sugar	14	Maize porridge with sugar	108	Maize porridge with sugar	108	Maize porridge with sugar	56
Cassava porridge with sugar	10	Maize porridge without sugar and milk	94	Maize porridge without sugar and milk	94	Mix flour porridge with sugar	55
Maize porridge without sugar and milk	9	Fish relish without oil	68	Fish relish without oil	68	Fish relish with oil	36
Cassava porridge (*Uji wa Muhogo*)	8	Mango, ripe, fresh-EP	60	Mango, ripe, fresh—EP	60	Fish relish without oil	36
Sorghum porridge with sugar	6	Cassava stiff porridge	55	Cassava stiff porridge	55	Cassava and red sorghum *ugali*	31
Sorghum porridge without sugar or milk	6	Maize sorghum *ugali* (stiff porridge)	54	Maize sorghum *ugali* (stiff porridge)	54	Mango, ripe, fresh-EP	28
Mango, ripe, fresh-EP	4	Fish relish with oil	54	Fish relish with oil	54	Maize sorghum *ugali* (stiff porridge)	25
Finger millet and sorghum porridge	4	Rice boiled with oil	43	Rice boiled with oil	43	Small dried fish with tomatoes and oil	25
Cassava porridge with milk and sugar	4	Small dried fish with tomatoes and oil	43	Small dried fish with tomatoes and oil	43	Cassava stiff porridge	24
Maize porridge with sugar and milk	3	Milk whole, 3.25% milkfat	40	Milk whole, 3.25% milk fat	40	Sweet potato, boiled	20
Fish relish without oil	3	Maize porridge with sugar and milk	40	Maize porridge with sugar and milk	40	Kidney bean relish with oil	19
Cassava flour, *ugali*	3	Cassava and sorghum porridge without sugar or milk	30	Cassava and sorghum porridge without sugar or milk	30	Tea without milk, with sugar	19
Mango juice	2	Kidney bean relish with oil	28	Kidney bean relish with oil	28	Maize porridge with sugar and milk	18
Millet porridge with sugar	2	Cassava and red sorghum *ugali*	27	Cassava and red sorghum *ugali*	27	Milk whole, 3.25% milk fat	17
Cassava stiff porridge	2	Tea without milk, with sugar	25	Tea without milk, with sugar	25	Cassava and maize *ugali*	17
Papaya, ripe	1	Fish, fresh, stew	24	Fish, fresh, stew	24	Cassava and sorghum porridge without sugar or milk	17
Maize *ugali* (stiff porridge)	1	Sweet potato, boiled	24	Sweet potato, boiled	24	Rice boiled with oil	15
Maize and sorghum *ugali* (stiff porridge)	1	Papaya, ripe	23	Papaya, ripe	23	Fish, fresh, stew	14
Beef relish with oil	1	Amaranth, leaves, picked, boiled, drained (without salt)	23	Amaranth, leaves, picked, boiled, drained (without salt)	23	Cassava, millet and sorghum porridge	14
Kidney bean relish with oil	1	Maize, porridge, milk	23	Maize, porridge, milk	23	Amaranth, leaves, picked, boiled, drained (without salt)	11
Kidney bean relish without oil	1	Cassava flour *ugali*	19	Cassava flour *ugali*	19	Yogurt, plain, whole milk	9
Cassava and red sorghum *ugali*	1	Cassava, millet and sorghum porridge	18	Cassava, millet and sorghum porridge	18	Green leaf, medium, relish with oil	9
Maize porridge milk	1	—	—	—	—	Cassava flour *ugali*	9

1 Numbers represent frequency out of total meals across all dietary recalls. EFFECTS, Engaging Fathers for Effective Child Nutrition and Development in Tanzania; EP, edible parts.

**FIGURE 3:**
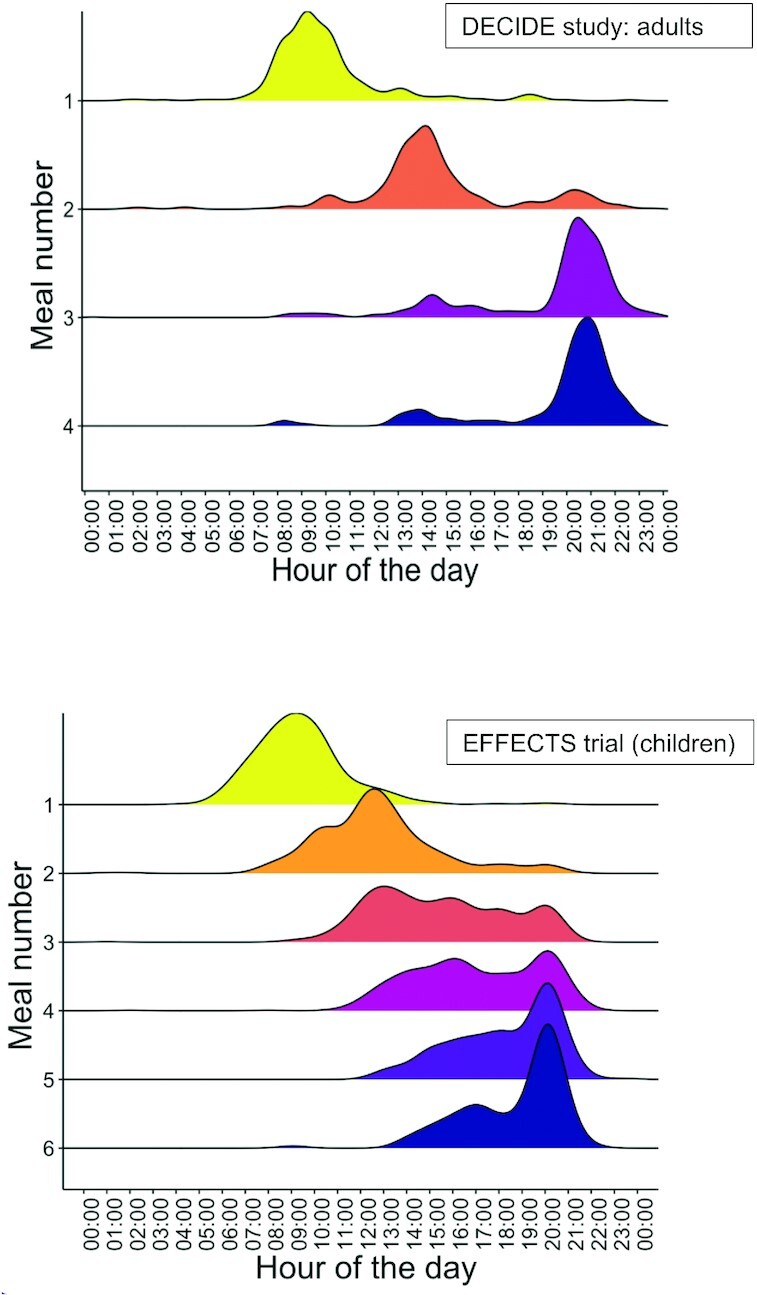
Timing of meals in DECIDE study (PLHIV adults; top panel) and EFFECTS trial (children <2 y of age, non–breast-milk foods; bottom panel). DECIDE, Diet, Environment, and Choices of positive living; EFFECTS, Engaging Fathers for Effective Child Nutrition and Development in Tanzania; PLHIV, persons living with HIV.

**Figure 3** shows histograms on the timing of meals in the DECIDE study and EFFECTS trial (non–breast-milk foods). Meals were grouped by timing—that is, 2 food items and a beverage consumed simultaneously were still accounted for as 1 meal. Urban adults typically consumed 3 meals (IQR: 3, 4) at 09:00, 13:00–14:00, and between 20:00 and 21:00. Timing of meals outside the home revealed that these comprised mostly the morning and lunch meals (**Supplemental Figure 2**), which are consumed outside the home compared to evening meals. Among those who reported eating food away from home, men had a more significant share of the total energy intake (median: 54%) than women (median: 39%). Distribution of meal timings for children (EFFECTS trial) revealed that morning meals and evening meals were highly structured in a smaller time window compared with midday meals (i.e., less variability in the distribution of the timing among all children).

**Table 7** shows the summary of nutrient intakes by gender in the DECIDE study. Urban men consumed a median intake of 3040 kcal, while women consumed 2468 kcal. Timing of energy intake across meals and over weekdays, as shown in **Figure 4**, illustrates the differences by gender. For men, the first 2 meals typically had a higher proportion of daily energy consumption, compared with evening or late-night meals, except most notably on Fridays (which are considered a holiday for Muslims). The opposite trend was observed for women, whose first meal typically provided <400 kcal,  while a higher share of daily energy was consumed later in the day. More women reported consuming the fourth meal (*n *= 143) compared with men (*n *= 43). Gender differences in protein intake (especially from animal-source food), overall fat intake, and zinc were also observed. There were no differences observed in carbohydrates, fiber, calcium, iron, vitamin A, or folate intake. Median micronutrient intakes for iron (10% bioavailability) and zinc (moderate availability) met the UN Recommended Nutrient Intakes (RNIs) for respective age and sex groups (24). However, median calcium, vitamin A, and folate intakes remained below the UN RNI (24).

**TABLE 7:** Meidan nutrient intakes from the DECIDE study by gender (PLHIV adults)^1^

	Tablet-based estimates		
	Male (*n* = 77)	Female (*n* = 235)	Total (*n* = 312)
Energy, kcal	3040.7 (2090.9, 4351.5)	2468.7 (1732.6, 3445.3)	2607.0 (1775.6, 3632.8)
Protein, g	89.6 (44.7, 151.7)	63.5 (41.3, 106.5)	66.9 (42.2, 114.2)
Animal-source protein, g	35.3 (4.4, 95.9)	20.2 (0.0, 60.2)	26.7 (0.0, 65.5)
Fat, g	112.2 (50.1, 180.7)	73.9 (42.5, 131.6)	79.7 (43.9, 144.7)
Carbohydrates, g	407.3 (240.3, 484.9)	363.2 (239.7, 501.3)	377.8 (240.3, 488.8)
Fiber, mg	24.4 (19.1, 33.9)	24.8 (16.4, 34.9)	24.7 (16.9, 34.6)
Calcium, mg	311.0 (119.2, 960.4)	362.7 (167.6, 1342.2)	350.9 (156.6, 1201.7)
Iron, mg	13.3 (8.0, 18.6)	11.3 (7.7, 17.2)	11.4 (7.9, 17.8)
Zinc, mg	11.2 (6.3, 16.6)	8.7 (5.6, 13.0)	8.9 (5.9, 14.1)
Vitamin A, μg RE	259.1 (97.0, 909.0)	276.1 (72.6, 771.1)	260.8 (76.5, 811.2)
Folate, μg	298.4 (173.0, 455.9)	268.4 (159.5, 433.6)	281.1 (163.8, 440.4)

1 Values are medians (IQR) from tablet-based recall. DECIDE, Diet, Environment, and Choices of positive living; PLHIV, persons living with HIV; RE, retinol equivalents.

**Table 8** shows the summary of nutrient intakes in the EFFECTS trial. Children's intake of all nutrients increased with age, where median energy intake ranged from 170 to 600 kcal/d among children 0–24 mo of age. Median micronutrient intakes for calcium, iron, zinc, and vitamin A were below the UN RNI for children 9–24 mo of age (24). However, median intakes for folate exceeded the UN RNI with 35.6 μg/d compared with the breast-milk–adjusted UN RNI of 27.6 μg/d for children aged 9–11 mo (24).

**TABLE 8:** Median Nutrient intakes among children 0–25 mo of age from the EFFECTS trial^1^

	EFFECTS (children)			
	0–5 mo (*n* = 83)	6–12 mo (*n* = 360)	13–18 mo (*n* = 262)	19–25 mo (*n* = 140)
Energy, kcal	169.2 (84.6, 292.3)	500.4 (312.7, 732.5)	770.8 (554.0, 1137.2)	932.5 (591.5, 1262.2)
Protein, g	4.5 (2.3, 8.7)	11.4 (5.8, 20.3)	21.0 (14.1, 30.6)	23.8 (16.1, 32.7)
Animal-source protein, g	2.3 (0.0, 6.8)	4.3 (0.0, 11.0)	8.6 (2.6, 17.2)	9.2 (2.8, 17.7)
Fat, g	6.4 (1.8, 11.3)	12.9 (5.9, 22.7)	17.3 (9.8, 28.6)	19.8 (11.5, 31.5)
Carbohydrates, g	23.6 (9.5, 38.6)	78.3 (46.9, 116.4)	124.1 (78.9, 204.4)	158.4 (98.0, 220.3)
Fiber, mg	0.9 (0.0, 3.6)	6.5 (3.4, 10.0)	10.4 (6.1, 15.3)	11.4 (8.2, 17.3)
Calcium, mg	93.6 (46.8, 243.2)	148.4 (59.6, 472.3)	348.4 (98.2, 788.2)	404.6 (107.7, 889.9)
Iron, mg	0.5 (0.2, 1.0)	2.8 (1.4, 4.6)	5.2 (3.3, 7.2)	6.5 (4.3, 8.8)
Zinc, mg	0.6 (0.4, 1.1)	1.7 (0.9, 3.4)	3.5 (2.3, 5.0)	3.9 (2.4, 6.0)
Vitamin A, μg RE	39.5 (1.0, 59.2)	36.8 (2.9, 97.9)	91.0 (25.8, 257.6)	99.5 (38.7, 222.9)
Folate, μg	10.9 (7.0, 21.4)	35.6 (16.3, 64.6)	66.5 (40.4, 100.7)	78.5 (54.1, 118.9)

1 Values are medians (IQR). EFFECTS, Engaging Fathers for Effective Child Nutrition and Development in Tanzania; RE, retinol equivalents.

## Discussion

Here we have developed and describe a tablet-based dietary intake tool (TZ-24hr-DR) that is easy to administer in Tanzania to collect dietary information from adults and children in an urban and a rural setting. We streamlined the transformation of raw data into food groups, meal timings, macronutrients, and micronutrients by embedding the same meal codes as the FCTs. Use of the multiple-pass method (25) and a summary review feature at the end of 24-h recall aided in collecting accurate data. We were able to run real-time analysis of dietary data because the data were sent to the server within hours of data collection. Real-time analyses enabled us to identify issues such as extreme amounts or when “two mornings of dietary data” were collected (technically within 24 h, e.g., 08:00 yesterday to 08:00 today), and nonplausible or unknown ingredients, where immediate feedback was sent to the field with automated reports to remedy those errors. We found gender differences among adult PLHIV regarding location of food consumption, share of energy intakes within a day, and in nutrients.

We describe the timing and the location of meals and energy intake (among adults) and characterized the meal structures among an urban population in Tanzania. There is virtually no post-processing involved to examine the timing and location of meal consumption patterns. Greater consumption of food outside the home was reported by men (37%) than women (7%), and most of these occasions occurred in the morning and afternoon meals (Supplemental Figure 2). Overall, food consumption outside the home among DECIDE participants (15% in adults) was lower compared with urban studies done in Kenya and Ghana, where 77–81% reported eating out (26). These differences could be due to the sample and location (DECIDE is peri-urban compared with these studies that were in urban settings). Generally, there appeared to be a trend where men were eating out for 2 out of 3 meals ( breakfast and lunch outside the home), and men also reported a more significant share of total daily expenditure of food consumed outside the home compared with women, which is comparable to another study conducted in Tanzania (27). Taken together, this adds to the larger trend observed in urban African cities, where an estimated one-quarter of the urban population eat daily meals outside the home, and many of these include consumption of “ultra-processed prepared” foods, such as rice with sauce and potato fries, away from home along with the consumption of other ultra-processed foods such as sodas and African donuts (3). We also see these trends translate into the timing of energy intakes where men show greater energy intakes in the first 2 meals (480–510 kcal) compared with their later meals (315–450 kcal) (Figure 4). Future dietary studies among adults should evaluate gender differences in meals and energy consumed outside the home.

Nutrient estimates for the adult intakes from the TZ-24hr-DR were similar to a dietary validity study conducted in the same community (28). This study validated a food-frequency questionnaire (FFQ) with 2 paper-based 24-h recalls conducted in the same study setting among adults (28). The energy estimates from TZ-24hr-DR were closer to the FFQ estimates (mean of 2599 kcal), while estimates on macronutrient intake were between the FFQ and paper-based 24-h recall, especially protein intakes (28). Women's energy estimates from the TZ-24hr-DR (median: 2469 kcal) were also aligned with another FFQ study conducted among PLHIV pregnant women in peri-urban Dar es Salaam, Tanzania (median: 2532 kcal) (29). Mean folate, vitamin A, and iron ranges from the TZ-24hr-DR align more closely with the estimates from the paper-based 24-h recall (28). Zack and colleagues (28) concluded that the FFQ was able to yield good estimates of macronutrients and minerals but not vitamins. It appears that the TZ-24hr-DR tool is a good measure of macro- and and micronutrients for adults, given that all the micronutrients estimated from the tablet-based recall range were closer to the paper-based methods from Zack et al. conducted among adults from the same study setting. However, additional validation studies are needed to affirm these findings, especially for calcium intakes (28).

Rural children in the Mara region were introduced to semi-solid foods before 6 mo of age, which consisted of different mixed-grain and staple porridges such as sorghum, cassava, millets, and maize. The variety of green-leafy vegetables in children's diets generally increased with age. Only morning and evening meals appeared to be more structured (in terms of timing), perhaps reflecting the time use of caregivers and livelihoods. Children's micronutrient estimates were also comparable to another study conducted in rural Manyara, Tanzania, where paper-based 24-h recalls were collected from children 9–24 mo of age (30). In our study, we found very low intakes of vitamin A, calcium, and folate (especially among the younger age groups), and medium intakes of zinc and iron.

We compared children's energy intake with acceptable energy ranges identified by Htet et al. (13) and based on Dewey and Brown's (31) recommendations. The acceptable energy intake range for breastfed children is 100–600 kcal for 6–8-mo-old children, 200–750 kcal for 9–11-mo-old children, and 500–1100 kcal for 12–23-mo-old children (13, 31). Based on these ranges, 60.8% of the breastfed children (*n *= 682) and non-breastfed children (*n *= 153) met the acceptable energy intakes. These estimates were also on par with other tablet-based methods, which appear to have lower misreporting rates on energy intakes than paper-based recalls (13, 32). Closer examination of the children outside the acceptable energy intakes reveals that an additional 20% of breastfed children and 30% of non-breastfed children fall within 200 kcal of acceptable energy ranges. Further examination of sources of foods, market availability, feeding practices, and overall bioavailability of these micronutrients will be conducted in planned analyses to give a contextual detail on the local availability of micronutrient-rich foods.

This study adds a new tool (available in Harvard dataverse https://doi.org/10.7910/DVN/NEMC70), database of new recipes (*n *= 28, see Supplemental Table 1), and new portion-size estimates (*n *= 88) to estimate dietary intake rapidly. This tool is useful for estimating both macro- and micronutrients in Tanzania, is a flexible platform to collect dietary data for children and adults, and is a practical tool to conduct dietary recalls among multiple members of the household. Using the recipe feature, we collected 825 recipes (data not shown) from respondents that could be further analyzed to examine variations from the Tanzanian FCT. Although direct validation was not conducted, the estimates appear to be comparable to recent studies conducted in Tanzania.

Existing FCTs within the country were instrumental in developing this tool, linking datasets with real-time analysis. Out of 54 countries in Africa, there are only 21 with a food-composition database, of which 8 were recently updated in the last decade. Given the availability and use of the Kenyan FCT (2018) in this analysis, and more recently, the availability of the Malawi FCT (2019) (33), the tool could be expanded for use in East Africa. This adds to the growing number of electronic 24-h data-collection tools available in LMIC contexts (9) that could be tremendously useful in a variety of contexts. For example, this tool could be used to examine transition in food systems, availability of food, or timing and location of meals to understand meals consumed outside the home and other dietary risk factors, dietary surveillance, or more importantly, in the context of interventions, such as food-fortification programs.

## Supplementary Material

nzac015_Supplemental_File

## Data Availability

Data described in the manuscript and code will be made available upon request pending ethical and study team approvals. Tanzanian dietary tablet collection tool is available on Harvard dataverse for research purposes (https://doi.org/10.7910/DVN/NEMC70). For further information, please contact rambikap@purdue.edu.

## Notes

This work was supported, in whole or in part, by the Bill & Melinda Gates Foundation [Grant Number ID: OPP1110043]. Under the grant conditions of the Foundation, a Creative Commons Attribution 4.0 Generic License has already been assigned to the Author Accepted Manuscript version that might arise from this submission.

This paper was presented at the American Society of Nutrition (ASN) in Baltimore, MD, USA (2019).

The Diet, Environment, and Choices of positive living (DECIDE): Evaluating personal and external food environment influences on diets among PLHIV (persons living with HIV) and families in Dar es Salaam, Tanzania, study is funded through the Drivers of Food Choice Grants Program by the Bill and Melinda Gates Foundation (ID: OPP1110043) and the Foreign, Commonwealth, and Development Office (FCDO). The EFFECTS trial is funded by the Eleanor J Crook Foundation and the Conrad N Hilton Foundation.

Author disclosures: The authors report no conflicts of interest. The funders did not play a role in study design, implementation, analysis, or interpretation of the data.

Supplemental Figures 1 and 2, Supplemental Report 1, and Supplemental Table 1 , and are available from the “Supplementary data” link in the online posting of the article and from the same link in the online table of contents at https://academic.oup.com/cdn/.

The DECIDE study is a collaborative project led by Purdue University, University of Chicago at Illinois, Muhimbili University, and Africa Academy of Public Health.

Abbreviations used: DECIDE, Diet, Environment, and Choices of positive living; EFFECTS, Engaging Fathers for Effective Child Nutrition and Development in Tanzania; FCT, food-composition table; FFQ, food-frequency questionnaire; IRB, Institutional Review Board; LMIC, low- and middle-income country; ODK, Open Data Kit; PLHIV, persons living with HIV; RNI, Recommended Nutrient Intake; TZ-24hr-DR, Tanzania 24-h recall tool.
