# Supplementary material for: Gender and Age Differences in Meal Structures, Food Away from Home, Chrono-Nutrition, and Nutrition Intakes among Adults and Children in Tanzania Using a Newly Developed Tablet-Based 24-Hour Recall Tool
Source: Curr Dev Nutr. 2022 Feb 8;6(3):nzac015. doi: 10.1093/cdn/nzac015 (PMC8929990; doi:10.1093/cdn/nzac015)
Supplement: nzac015_Supplemental_File [file nzac015_supplemental_file.docx]

**Online supplementary materials:**

**Figure 1: Photo of interview aids (Photo taken by Stella Nyamsangia and approved for publication, independent researcher)**


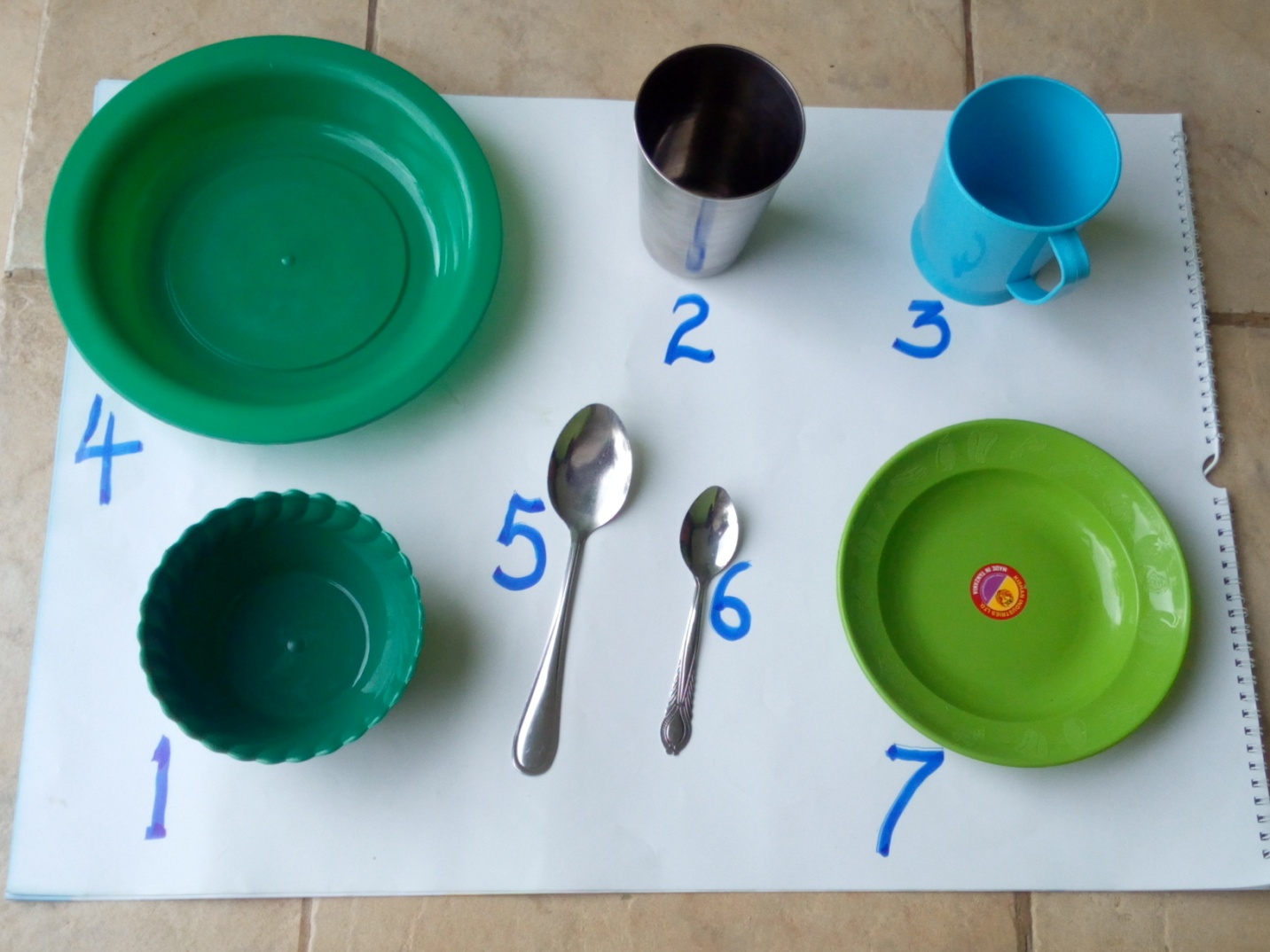


**Report 1: Sample daily reports and individual dietary profiles for real time monitoring.**

# Effects Food Recall (24 hours) Summary

The summary includes data from 24 hour recall.

This report was created on "11:05:43, 9 Jan 2019".

So far, 24 hour recall has been collected on 209 children.

Overall, 194 children are breastfed. Yesterday, range of children's breastfeeding frequency goes from 2 to 19 with the median breastfeeding frequency of 5 .


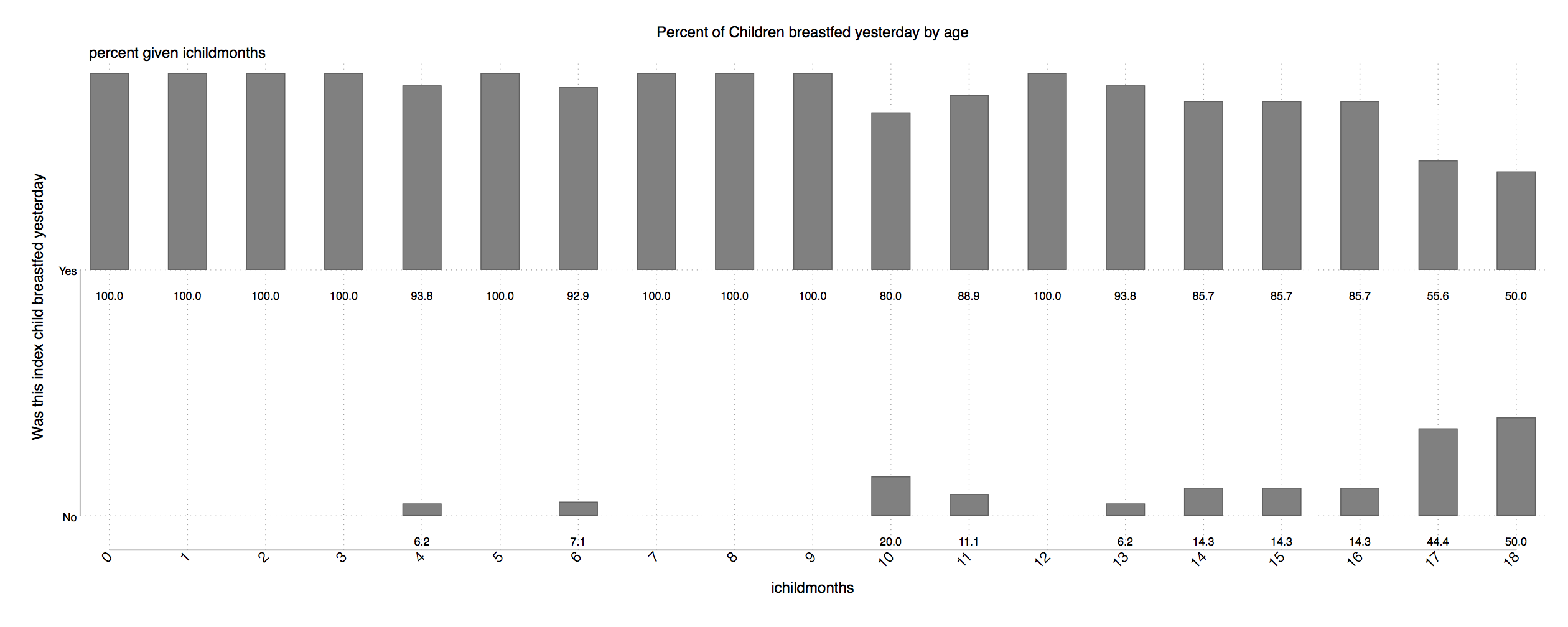


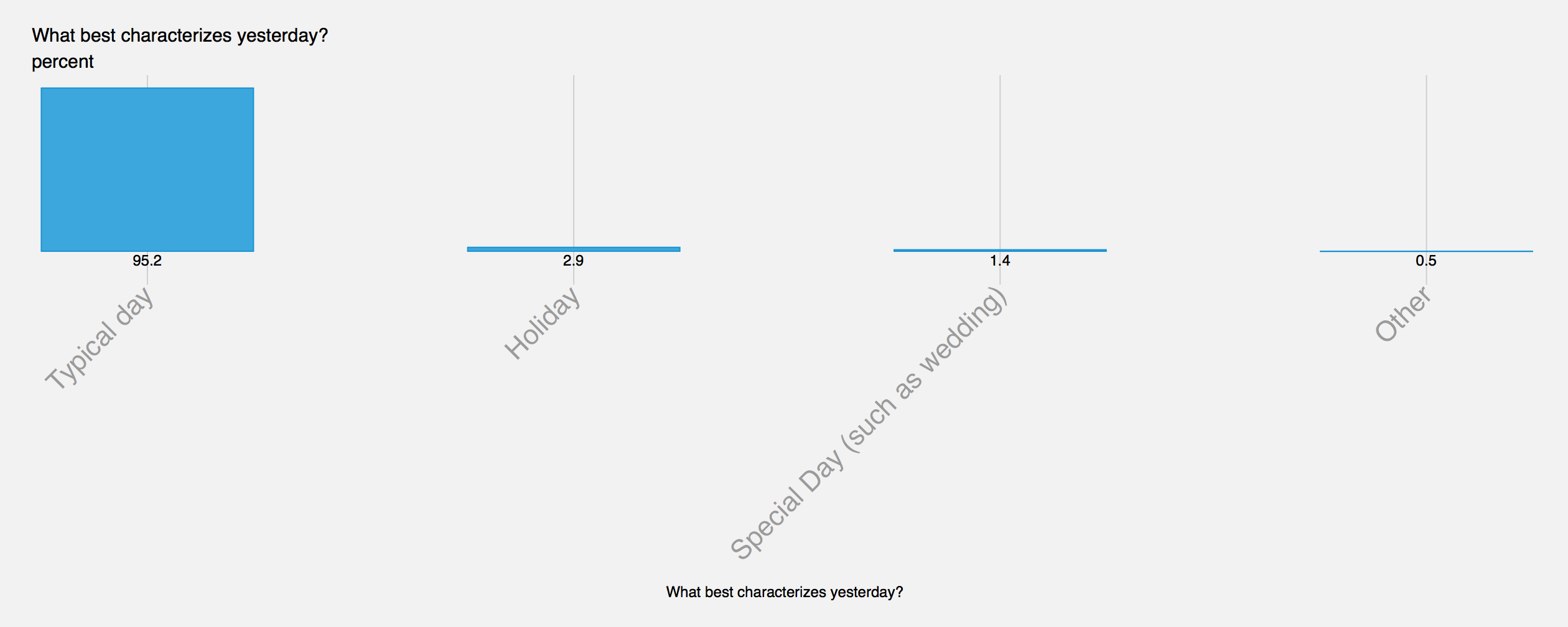


Overall, 179 children consumed non-breastmilk foods. Yesterday, range of children's meal frequency (including breastmilk) goes from 2 to 17 with the median frequency of 8.

Yesterday, range of children's meal frequency (not including breastmilk) goes from 0 to 9 with the median meal frequency of 3.


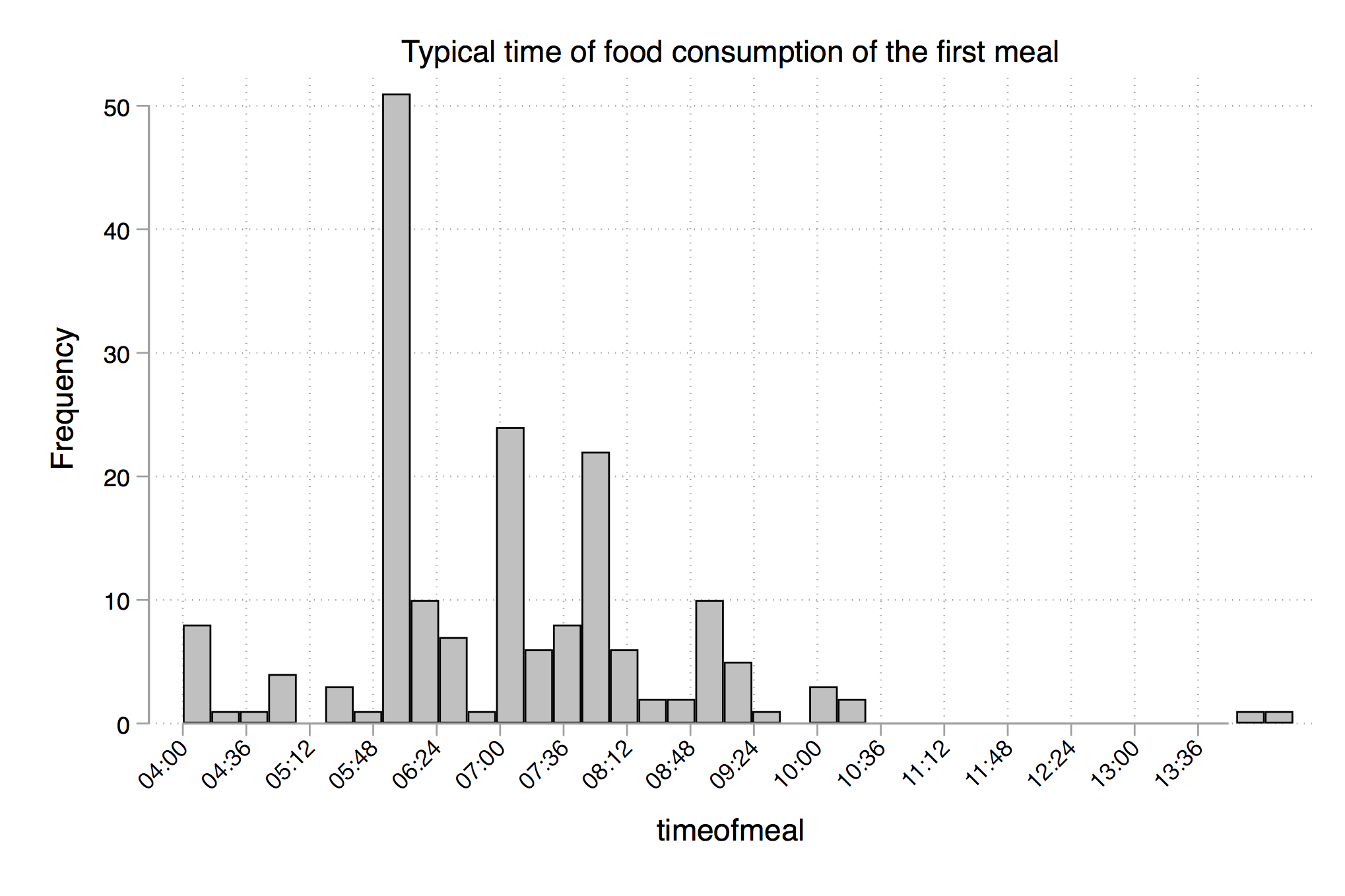


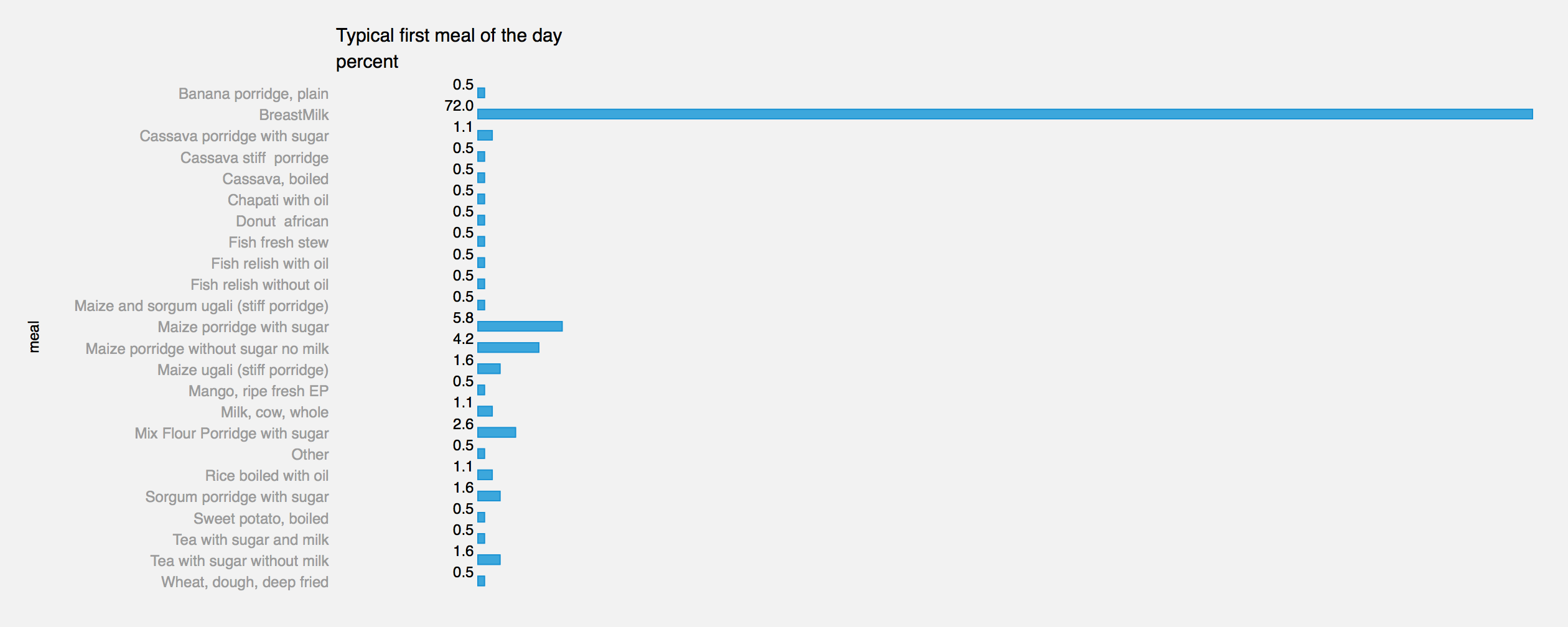


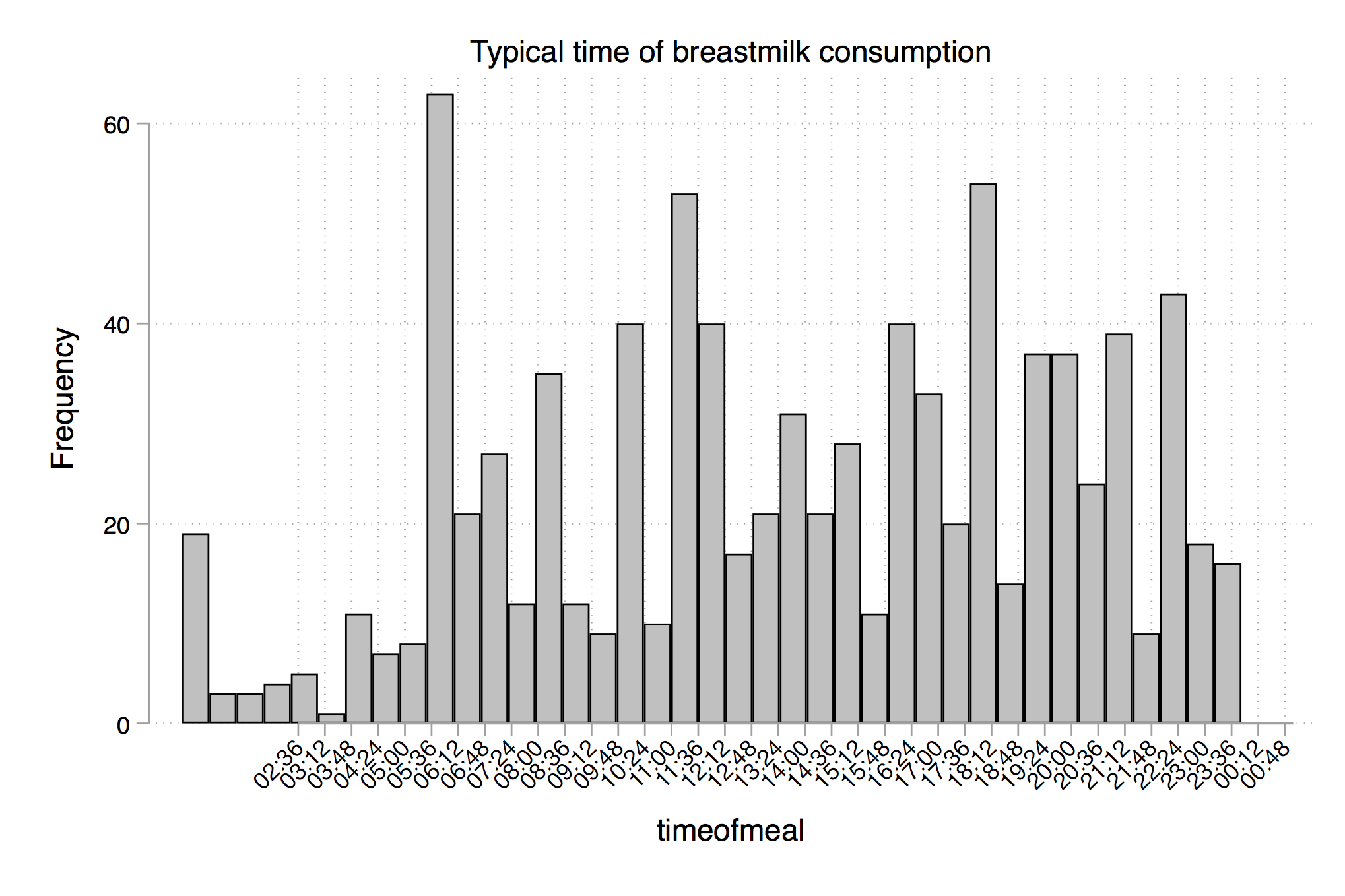


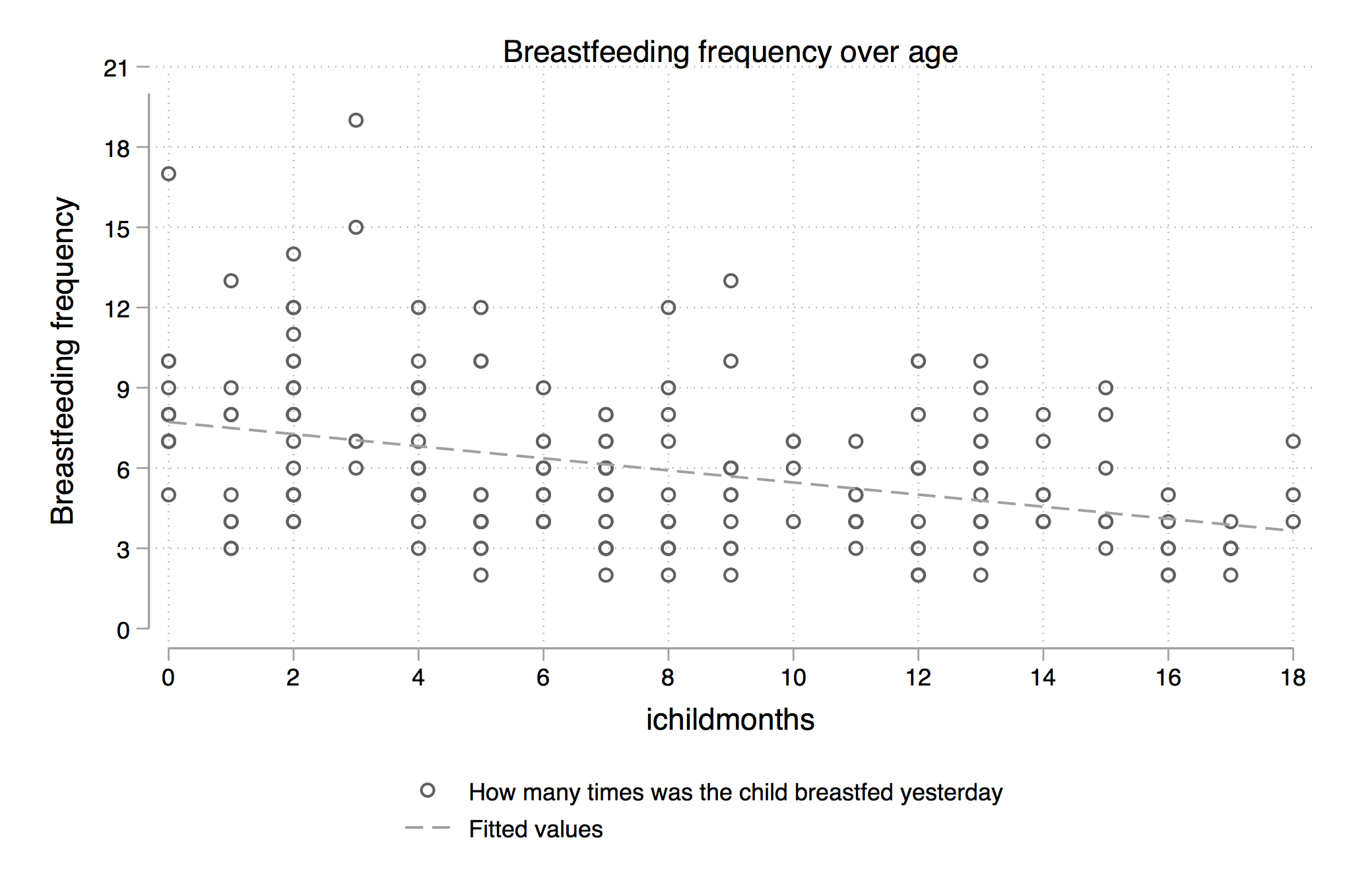


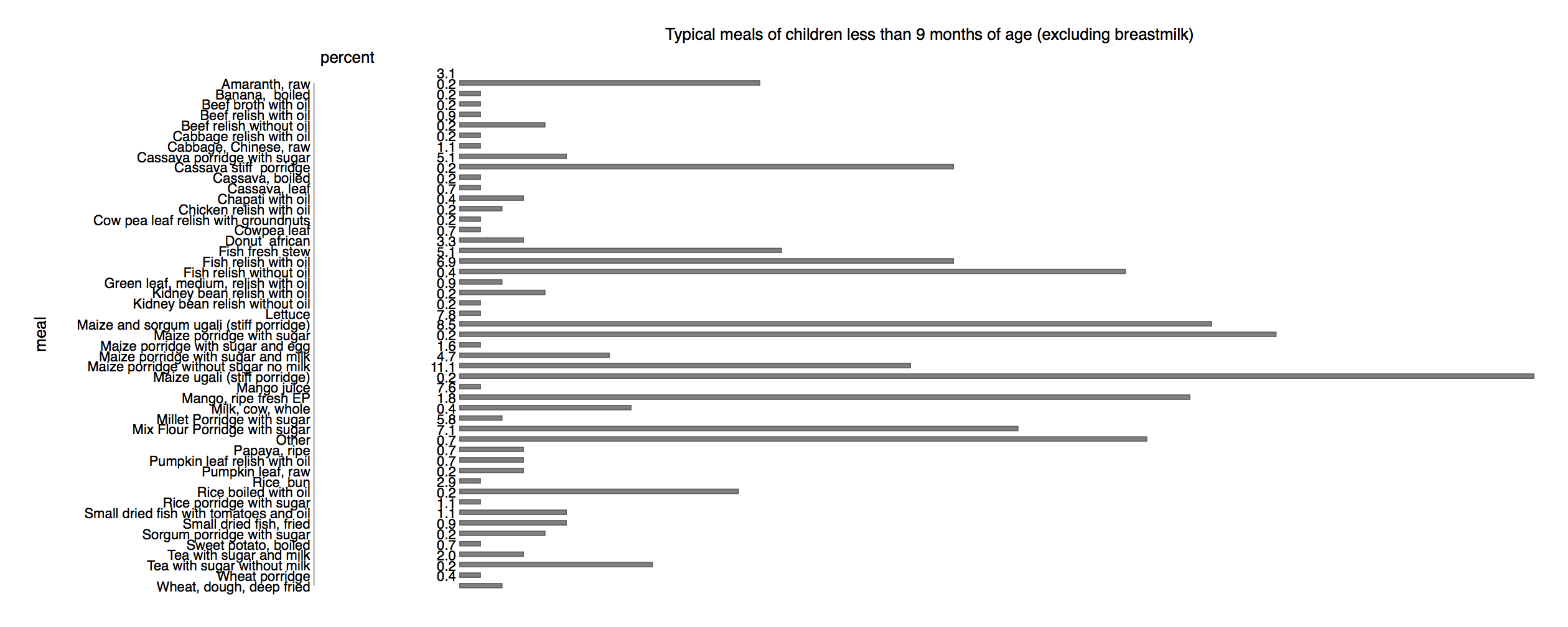


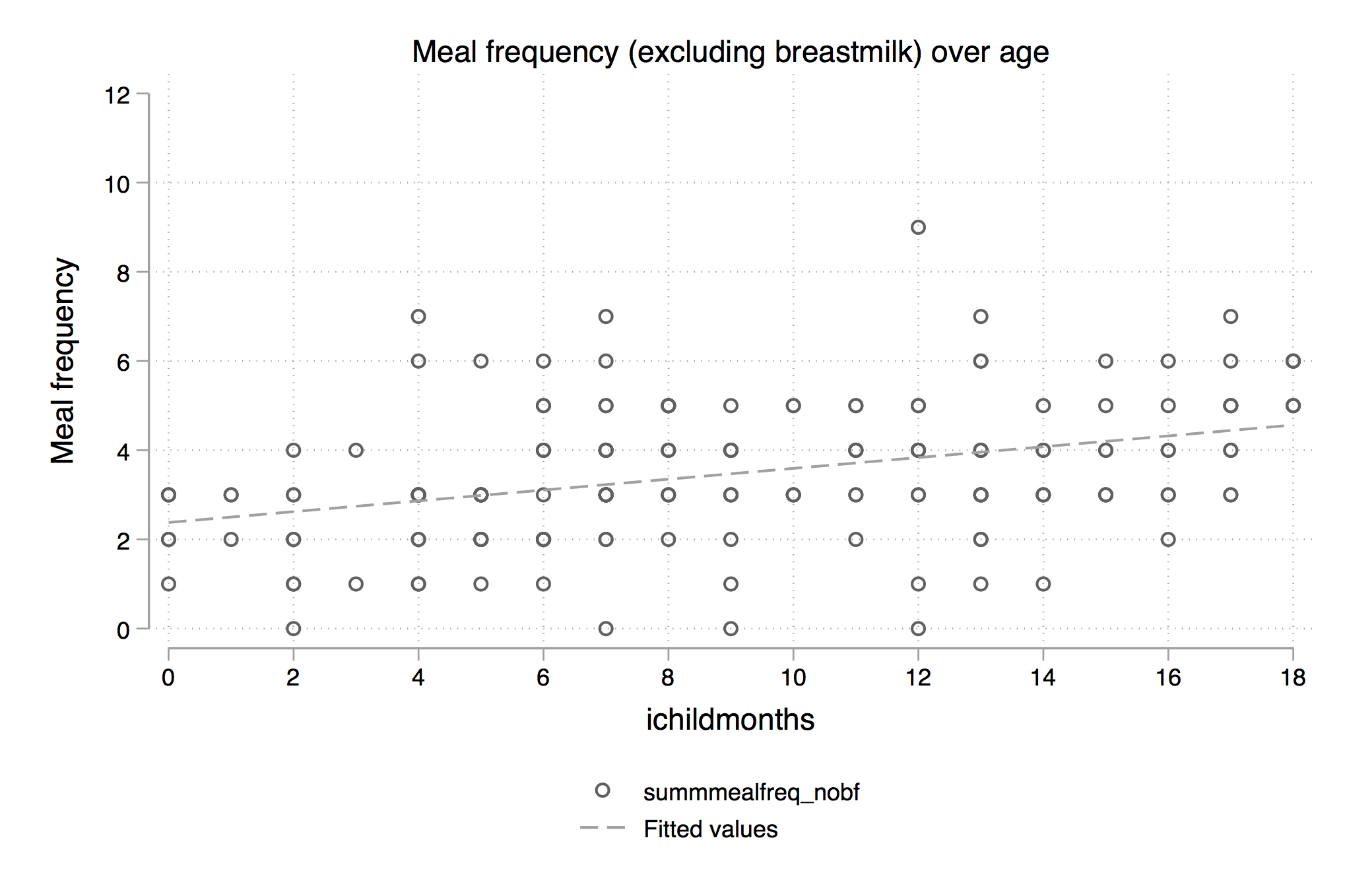


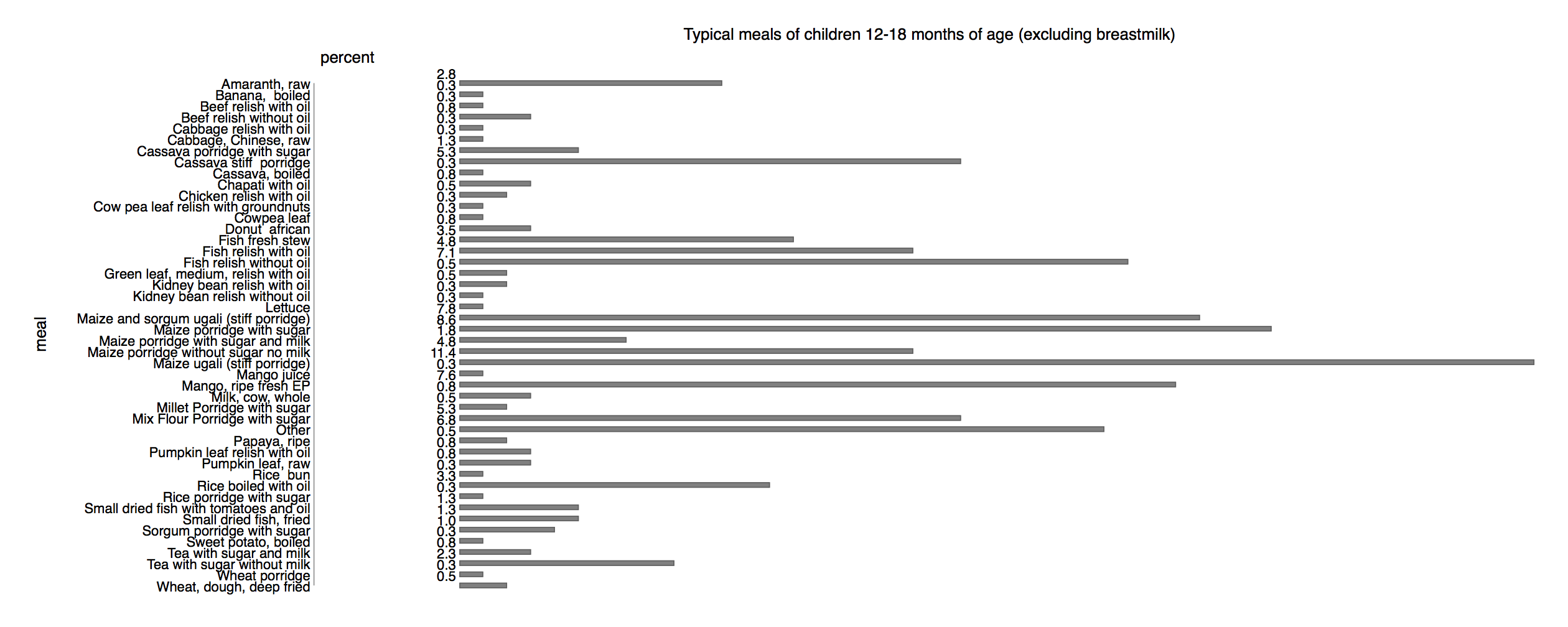


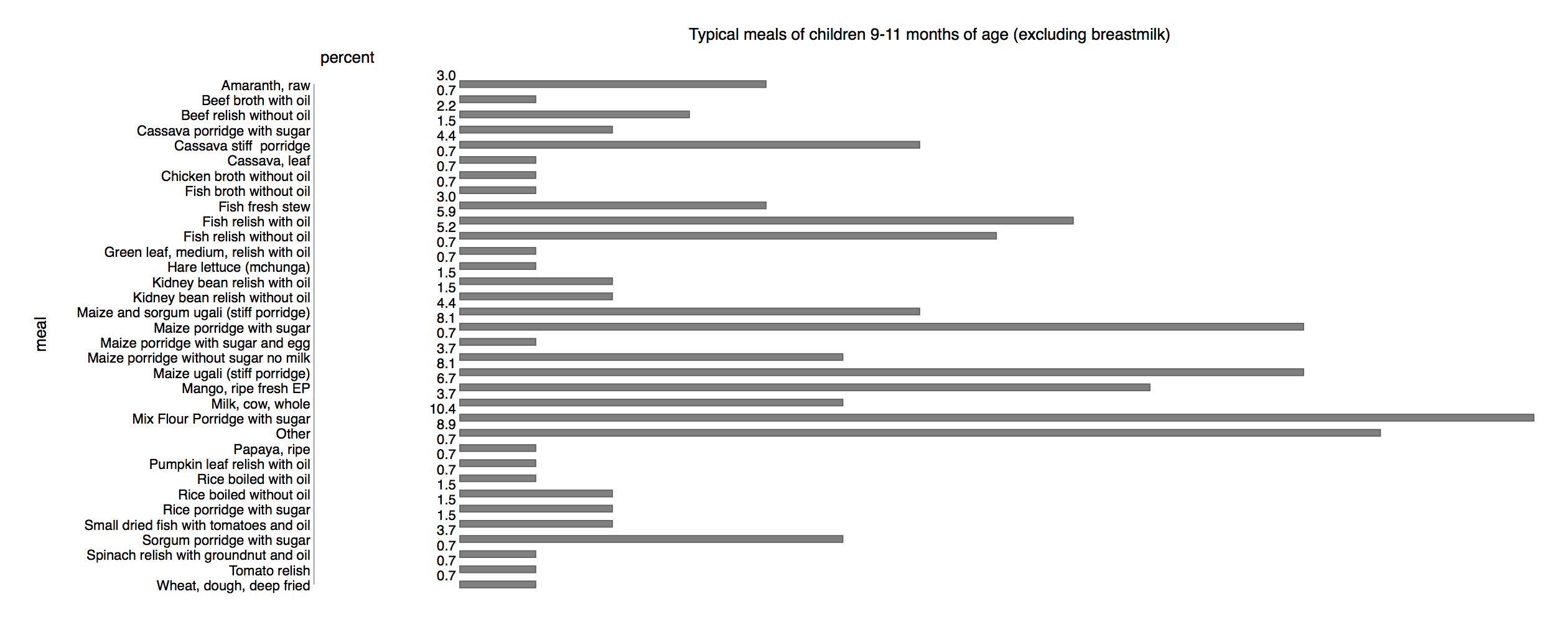


**Table 1: List of new recipes developed from both Effects and DECIDE.**

| **Assigned code** | **Meal/recipe names** |
| --- | --- |
| 20001 | Octopus with tomato and onion |
| 20002 | Octopus fish stew |
| 20003 | Banana fish stew |
| 20004 | Cow tripe stew |
| 20005 | Banana potato stew |
| 20006 | Cowpea relish with milk |
| 20007 | Kidney bean relish with milk |
| 20008 | Cassava porridge with milk and sugar |
| 20009 | Maize and sorghum porridge with milk and sugar |
| 20010 | Maize and sorghum porridge with milk only |
| 20011 | maize and sorghum porridge with only sugar |
| 20012 | stiff maize and sorghum porridge |
| 20013 | Cassava and maize ugali |
| 20014 | Maize porridge with milk but no sugar |
| 20019 | Millet porridge with sugar and milk |
| 20016 | Cassava and sorghum porridge with milk only |
| 20017 | Cassava and sorghum porridge without sugar or milk |
| 20018 | Cassava and sorghum porridge with milk and sugar only |
| 20008 | Cassava porridge with milk and sugar |
| 20020 | Rice cooked with palm oil |
| 20021 | Rice cooked with palm oil and milk |
| 20022 | Rice cooked with milk |
| 20023 | Small, dried fish relish with cow milk |
| 20024 | Small, dried fish relish tomatoes with palm oil |
| 20025 | Small, dried fish relish without oil |
| 20026 | Sorghum porridge with milk but no sugar |
| 20027 | Sorghum porridge with milk and sugar |
| 20028 | Sorghum porridge without milk or sugar |

**Figure 2: Timing of meals consumed outside the home from DECIDE study:**
